# Fast quantitative MRI: Spiral Acquisition Matching-Based Algorithm (SAMBA) for Robust T_1_ and T_2_ Mapping

**DOI:** 10.1016/j.jmro.2024.100157

**Published:** 2024-07-29

**Authors:** Mireia Perera-Gonzalez, Christina J. MacAskill, Heather A. Clark, Chris A. Flask

**Affiliations:** a Department of Bioengineering, Northeastern University, Boston MA, USA; b Department of Radiology, Case Western Reserve University, Cleveland OH, USA; c School of Biological and Health Systems Engineering, Arizona State University, Tempe AZ, USA; d Departments of Biomedical Engineering and Pediatrics, Case Western Reserve University, Cleveland OH, USA

**Keywords:** Quantitative MRI, Spiral Acquisition Matching-Based Algorithm (SAMBA), low-field preclinical MRI

## Abstract

Conventional diagnostic images from Magnetic Resonance Imaging (MRI) are typically qualitative and require subjective interpretation. Alternatively, quantitative MRI (qMRI) methods have become more prevalent in recent years with multiple clinical and preclinical imaging applications. Quantitative MRI studies on preclinical MRI scanners are being used to objectively assess tissues and pathologies in animal models and to evaluate new molecular MRI contrast agents. Low-field preclinical MRI scanners (≤3.0T) are particularly important in terms of evaluating these new MRI contrast agents at human MRI field strengths. Unfortunately, these low-field preclinical qMRI methods are challenged by long acquisition times, intrinsically low MRI signal levels, and susceptibility to motion artifacts. In this study, we present a new rapid qMRI method for a preclinical 3.0T MRI scanner that combines a Spiral Acquisition with a Matching-Based Algorithm (SAMBA) to rapidly and quantitatively evaluate MRI contrast agents. In this initial development, we compared SAMBA with gold-standard Spin Echo MRI methods using Least Squares Fitting (SELSF) in vitro phantoms and demonstrated shorter scan times without compromising measurement accuracy or repeatability. These initial results will pave the way for future in vivo qMRI studies using state-of-the-art chemical probes.

## 1. Introduction

Magnetic Resonance Imaging (MRI) is a noninvasive imaging modality of interest for a wide range of clinical applications due to its high spatial resolution and soft-tissue contrast [1]. While traditional MRI relies on qualitative interpretation by radiologists [2], quantitative MRI (qMRI) provides an objective assessment of tissues and pathologies, crucial for understanding disease mechanisms and evaluating therapies. qMRI is particularly valuable in preclinical studies using animal models to develop new MRI probes and sensors targeting specific disease molecular processes. The magnetic field strength in preclinical MRI scanners typically range from 1.0T to 15.2T, presenting a tradeoff the higher the magnetic strength between improved resolution and reduced translational capabilities when exploring newer molecular MRI contrast agents. The application of qMRI, especially in low-field preclinical scanners (≤3.0T), is hindered by (1) long scan times, particularly with conventional spin-echo MRI protocols to estimate T_1_ and T_2_ relaxation times [3]; and (2) susceptibility to respiratory motion artifacts, which require gating that can further extend the acquisition time. Conventional faster MRI approaches, such as echo-planar imaging (EPI) and balanced Steady-State Free Precession (bSSFP) come at the cost of increased eddy current and banding artifacts, respectively [4,5]. Therefore, there is a critical need for new, rapid, motion-resistant qMRI methods for low-field preclinical MRI scanners, particularly for evaluating new molecular MRI probes and sensors accurately and effectively with sufficient spatial and temporal resolution.

Herein, we propose a rapid preclinical qMRI method “SA-MBA” (<5 min/scan) to objectively evaluate contrast agents at 3.0T based on T_1_ and T_2_ maps. Inspired by the increasing developments in MR Fingeprinting [6] techniques, SAMBA combines a spiral acquisition “SA” (accelerates data sampling, decreasing scan durations) [5] and a dictionary matching-based algorithm “MBA” [1,6] (with inherent resistance to motion artifacts [7] in comparison to conventional least squared error fitting methods [3]). While the majority of components have been described previously, this is the first implementation of these spiral and MBA methods that we are aware of on a preclinical 3.0T MRI scanner [8]. In this study, the performance of SAMBA against gold-standard spin echo MRI methods has been evaluated with in vitro phantoms to pave the path for future development of fast, quantitative acquisitions for low-field MRI scanners.

## 2. Methods

Dedicated phantom samples (Supplemental Table 1) [9] were scanned at room temperature (22 ± 0.5 °C) in a preclinical 3.0T with a 30 mm volume mouse coil, running on Paravision 360 (Bruker Biospin, Billerica, MA). Scanning parameters for the T_1_ and T_2_ SAMBA and spin echo (SE) MRI scans are shown in Table 1. T_1_ SAMBA consisted of an adiabatic inversion preparation pulse [10] followed by 50 successive excitations and spiral readouts every 265 milliseconds (5 interleaves, 3535 data points/interleaf). T_2_ SAMBA consisted of a variable-length 90°+180°preparation schema [10] followed by a single spiral acquisition (5 interleaves, 11,393 data points/interleaf). A reference phantom (2 % agarose, 50 ml falcon tube) was used for spiral trajectory measurements for improved image reconstruction. Spin Echo T_1_ images were acquired at 13 inversion times: 26, 79, 265, 530, 790, 1050, 1320, 1590, 1860, 2120, 3970, 6610, 9250 ms. Spin Echo T_2_ images were acquired at 7 T_2_ preparation times: 12, 25, 50, 75, 100, 200, 400 ms [11] Each scan protocol was repeated 8 times in succession for repeatability assessment.

All images were exported for offline analysis and calculation of T_1_ and T_2_ maps in MATLAB (MathWorks, Natick, MA). Established exponential decay models were applied to the magnitude images [12,13], and regions-of-interest (ROI) were defined on each image to obtain the average values. Spin Echo T_1_ and T_2_ maps were obtained with conventional linear least squared error fitting. SAMBA T_1_ and T_2_ maps were derived from a dedicated dictionary as previously described [14,15] (Supplemental Figure 1).

Statistical analysis was performed via GraphPad Prism (8.4.3). Statistical significance was defined at *p*< 0.05. Linear regression analysis, Bland-Altman plots, and two-tailed Student’s *t*-tests were performed to compare the T_1_ and T_2_ estimates between SAMBA and SELSF methods. The repeatability for each method was determined by calculating the coefficient of variation (CV% = standard deviation / mean * 100 %) for the 8 consecutive scans [16]. The CV% * scan time (in hours) to account for the different acquisition times.

## 3. Results

### 3.1. Accuracy and precision

**Fig. 1.**
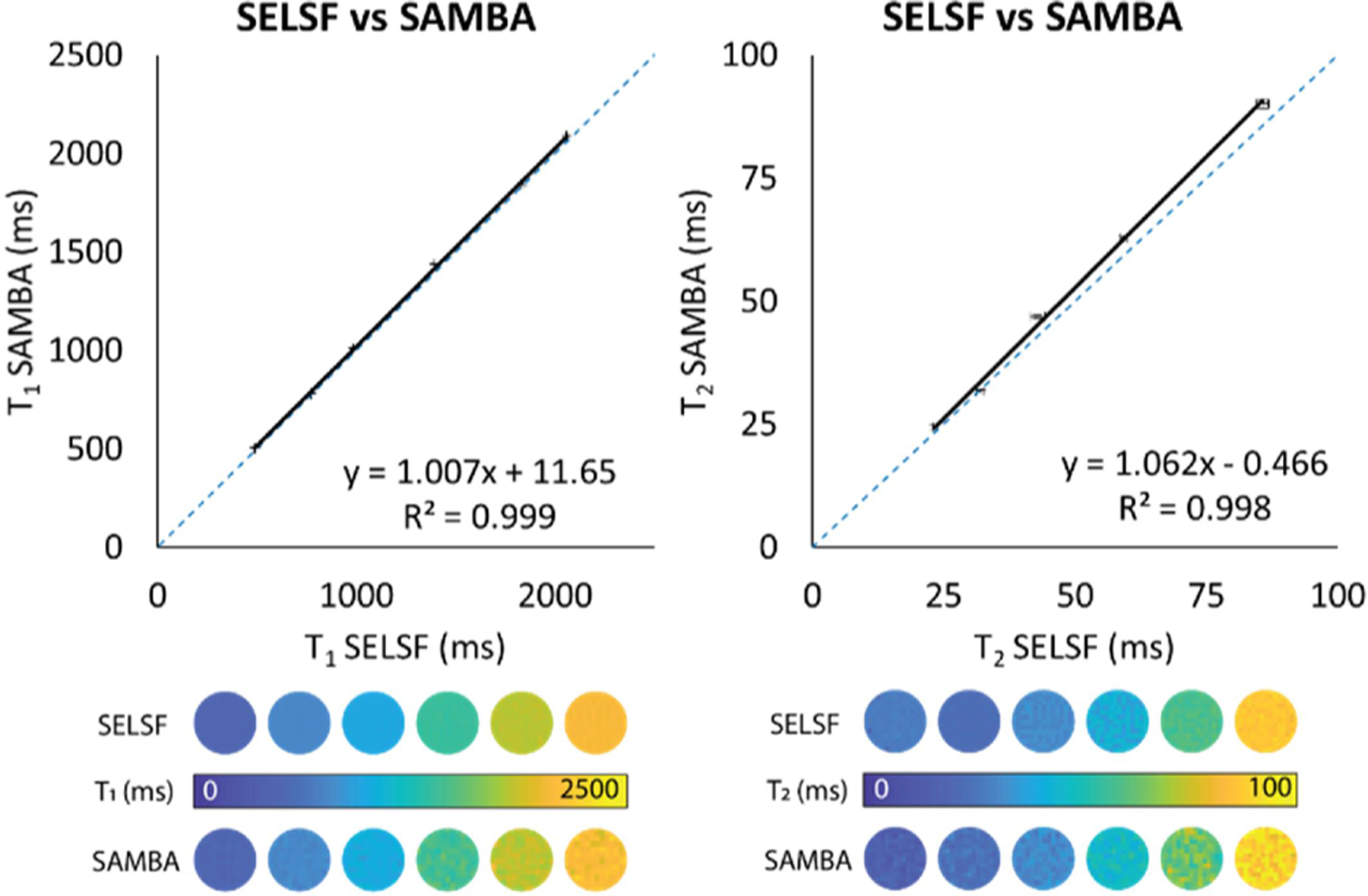
Linear regression and representative colormaps of T_1_ (left) & T_2_ measurements (right) obtained via conventional approach (Spin Echo Least Squares Fitting “SELSF” x axes) vs. proposed method (Spiral Acquisition Matching-Based Algorithm “SAMBA”, y axes). Dashed line represents the unity, error bars represent standard deviation between 8 measurements.

Representative T_1_ and T_2_ maps and linear regression for SAMBA and SELSF are shown in Fig. 1. A strong linear correlation was demonstrated between SAMBA and SELSF results over 8 measurements (T_1_: R^2^ = 0.999, slope = 1.007, y-intercept = 11.65 ms; T_2_: R^2^ = 0.998, slope = 1.062, y-intercept = −0.466 ms).

SAMBA and SELSF results were highly correlated despite a slight overestimation for T_2_, indicating that SAMBA can accurately calculate T_1_ and T_2_ maps. Beyond comparing SELSF and SAMBA, all possible permutations were analyzed (Supplemental Figs. 2 & 3) with less than 2 % deviation from the unity except for Spin Echo LSF vs. Spiral Acquisition LSF (T_2_: R^2^ = 0.999, slope = 1.058, y-intercept = 1.935 ms), and Spin Echo MBA vs. Spiral Acquisition MBA (T_2_: R^2^ = 0.999, slope = 1.075, y-intercept = 0.179 ms).

### 3.2. Repeatability measurement

**Fig. 2.**
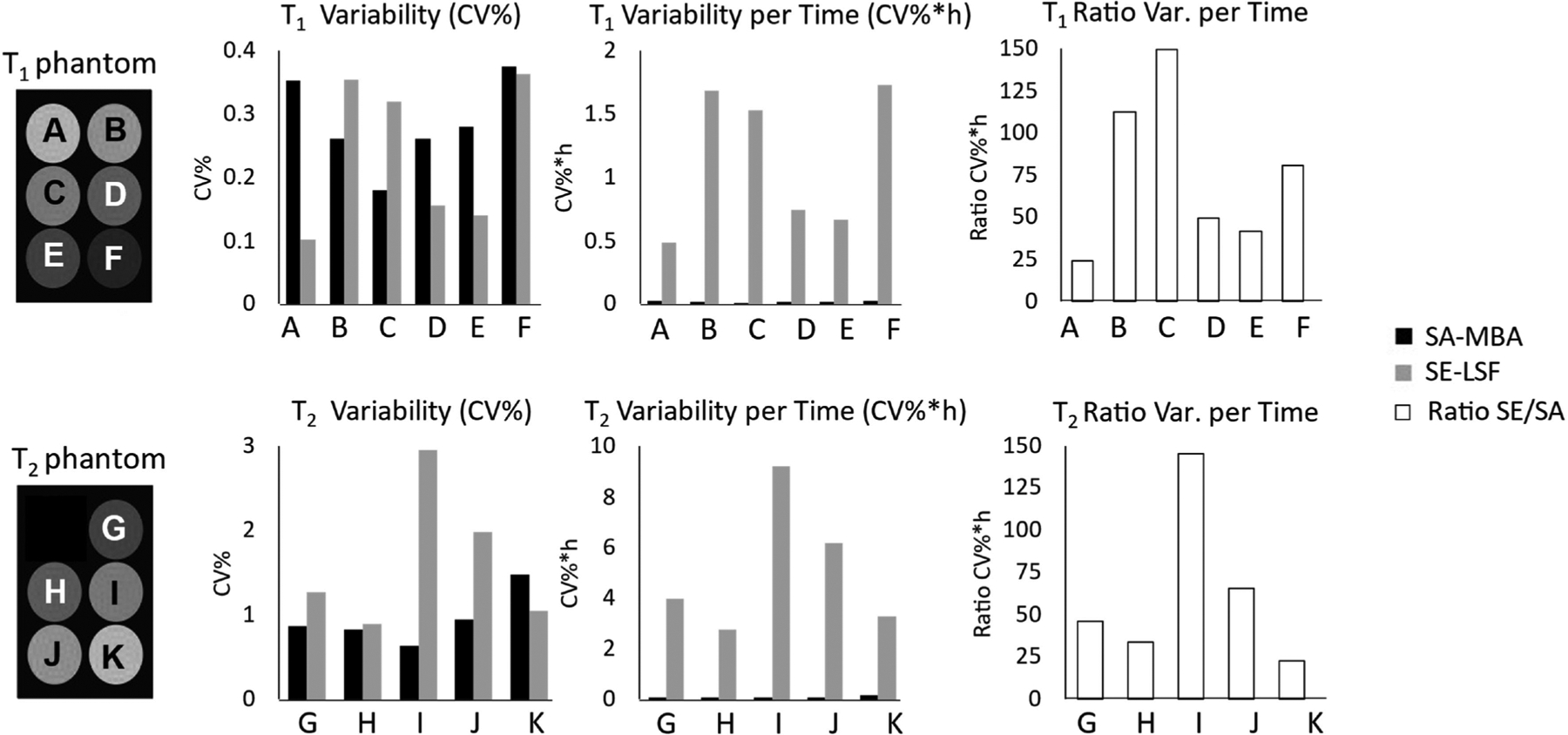
Analysis of measurement repeatability from T_1_ & T_2_ phantoms (left column): comparison of Coefficient of Variation (CV%) and CV% per time (middle columns), and ratio between conventional and proposed approaches (right column).

Measurement variability and variability per time was obtained from the Coefficient of Variation CV% over 8 measurements (Fig. 2). On average, variability (CV%) was 0.30 ± 0.10 % for T_1_ SAMBA, 0.26 ± 0.14 % for T_1_ SELSF, [16] 0.95 ± 0.32 % for T_2_ SAMBA, and 1.63 ± 0.85 % for T_2_ SELSF. The low CV% for both T_1_ and T_2_ SAMBA (<1 %) highlights the faster mapping achieved by SAMBA without compromising repeatability. SAMBA resulted in ~20–150x increase in repeatability per time when considering acquisition time (CV%*hour). Agreement between the SAMBA and SELSF was further investigated via Bland-Altman analysis [16,17], Supplemental Figure 4): mean T_1_ bias = 20.82 ms, 95 % limits of agreement (LOA) = [−6.31, 47.96] ms; and mean T_2_ bias = 2.56 ms, 95 % limits of agreement (LOA) = [−1.49, 6.61] ms).

## 4. Discussion

This study introduces a fast, quantitative MRI sequence [13] for T_1_ and T_2_ Spiral Acquisition with a Matching-Based Algorithm (SAMBA) implemented in a preclinical 3.0T scanner [6]. Developing successful fast qMRI approaches for in vivo [10] preclinical studies at low-field is crucial to better understand molecular processes [16] evaluate pathophysiology, and develop novel chemical probes or sensors.

We, therefore, present SAMBA as a fast, accurate, and precise T_1_ and T_2_ mapping approach at low-field preclinical scanners, paving the path for easier translation of findings to clinical settings of similar field strengths [16].

SAMBA produced T_1_ and T_2_ maps significantly faster but strongly correlated with gold-standard Spin Echo Least Squares Fitting “SELSF” approaches (linear associations for T_1_: R^2^ = 0.999, slope = 1.007, y-intercept = 11.65 ms; T_2_: R^2^ = 0.998, slope = 1.062, y-intercept = −0.466 ms; Fig. 1 and Supplemental Figures 2 & 3). T_1_ SAMBA and SELSF results had a stronger correlation than those for T_2_, which has been previously attributed to a heavier influence of B_1_ inhomogeneities for T_2_ sequences implementing similar matching-based approaches [16]. Although B_1_ map correction [18], slice profile, and B1^+^compensation [19] approaches are beyond the scope of this study, their implementation could serve to increase the accuracy of T_2_ estimates in future work [20–22]. Both SAMBA and SELSF had comparable repeatability (T_1_: 0.30 ± 0.10 % SAMBA vs. 0.26 ± 0.14 % SELSF; T_2_: 0.95 ± 0.32 % SAMBA vs. 1.63 ± 0.85 % SELSF). SAMBA has the most positive impact when analyzing the decrease in variation CV% per scan time (Fig. 2), in comparison to SELSF.

SAMBA performance demonstrated against SELSF defines a pathway towards the analysis of MRI sensors in vitro with high accuracy and repeatability even when using low volumes and concentrations [23], therefore advancing the field of MRI for chemical imaging [24,25]. Future studies should investigate the performance of SAMBA as a preclinical in vivo imaging platform [2] to reduce sensitivity to motion artifacts [26]. In vivo SAMBA imaging could serve to quantify biomarkers, characterize treatment responses, and monitor chemical signals (e.g., neurotransmitter Acetylcholine) [27].

At the sequence level, future projects could optimize SAMBA by implementing parallel imaging^27^or undersampling methods to improve temporal performance for dynamic contrast-enhanced MRI studies, although they lead to signal-deprived images. Increasing coverage via 3D acquisitions could compensate for this by increasing the signal, although at the cost of extended scan times that may limit future in vivo dynamic MRI studies using molecular MRI contrast agents. In addition, the sequence could also characterize other parameters (diffusion, non-contrast perfusion, magnetization transfer [28], and chemical exchange saturation transfer [29]) by adjusting the preparation schema [8]. Lastly, SAMBA measurements could be analyzed via semi-automatic approaches based on artificial intelligence frameworks [30], providing further potential improvements in time efficiency, cost, and accuracy of existing practice.

In summary, we have reported and evaluated a novel quantitative MRI sequence specifically designed for a preclinical 3.0T MRI scanner based on a spiral trajectory scheme and a matching-based algorithm (SAMBA), resulting in much shorter scan times without compromising the accuracy or repeatability in T_1_ and T_2_ estimates in comparison to conventional spin echo MRI [16]. This SAMBA platform could pave the way for more advanced in vivo non-invasive molecular quantitative methods, facilitating the development and evaluation of state-of-the-art chemical probes and serving as a foundation to further advance the study of biochemical pathways and quantitative biomarkers non-invasively [13], and ultimately unlock the full potential of MRI for chemical imaging of living systems. Future lines of research could explore multi-site, multi-strength, and multi-vendor quantifications in efforts to standardize the measurements for quantitative in vivo MRI [2].

## Supplementary Material

1

## Figures and Tables

**Table 1:** Sequence parameters for spiral and spin echo.

Parameter	SAMBA T_1_	Spin Echo T_1_	SAMBA T_2_	Spin Echo T_2_
**TR (ms)**	14,000	10,000	5000	5000
**TE (ms)**	1.66	3.61	–	–
**TP (ms)**	–	–	12, 25, 50, 75, 100, 200, 400	12, 25, 50, 75, 100, 200, 400
**nTI**	50	13	–	–
**FOV (mm)**	30 × 30	30 × 30	30 × 30	30 × 30
**MS**	128 × 128	128 × 128	160 × 160	160 × 160
**ST (mm)**	1	1	1	1
**FA (°)**	180,20	180,90	90	90,180
**NSA**	3	3	2	2
**Scan time**	3min30s	4h45min	6min	3h

Abbreviations: TR = repetition time, TE = echo time, TP = preparation time, nTI = number of inversion times, FOV = field-of-view, MS = matrix size, ST = slice thickness, NSA = number of signal averages, FA = flip angle.

## Data Availability

Data will be made available on request.
