# Supplementary material for: Fast quantitative MRI: Spiral Acquisition Matching-Based Algorithm (SAMBA) for Robust T1 and T2 Mapping
Source: J Magn Reson Open. Author manuscript; Available in PMC 2024 Sep 25. (PMC11423800; doi:10.1016/j.jmro.2024.100157)
Supplement: 1 [file NIHMS2022763-supplement-1.docx]

# Supplementary information: TABLES & FIGURES

Table 1 Expected T_1_ and T_2_ values for phantoms at different concentrations of NiCl_2_, based on Stupic et al (2021)’s publication. Values in bold were used for the experiments in this work.

| Phantom | A | B | C | D | E | F | G | H | I | J | K |
| --- | --- | --- | --- | --- | --- | --- | --- | --- | --- | --- | --- |
| **ICP **NiCl_2_** (mm)** | 0.17 | 0.27 | 0.56 | 1.05 | 1.68 | 2.58 | 2.58 | 11.7 | 16.8 | 23.8 | 33.9 |
| **T_1_ @3T (ms)** | **2250** | **1990** | **1450** | **984** | **706** | **497** | 126 | 89.0 | 62.7 | 44.5 | 30.8 |
| **T_2_ @3T (ms)** | 1650 | 1470 | 1080 | 718 | 510 | 360 | **90.3** | **64.3** | **45.7** | **31.9** | **22.4** |


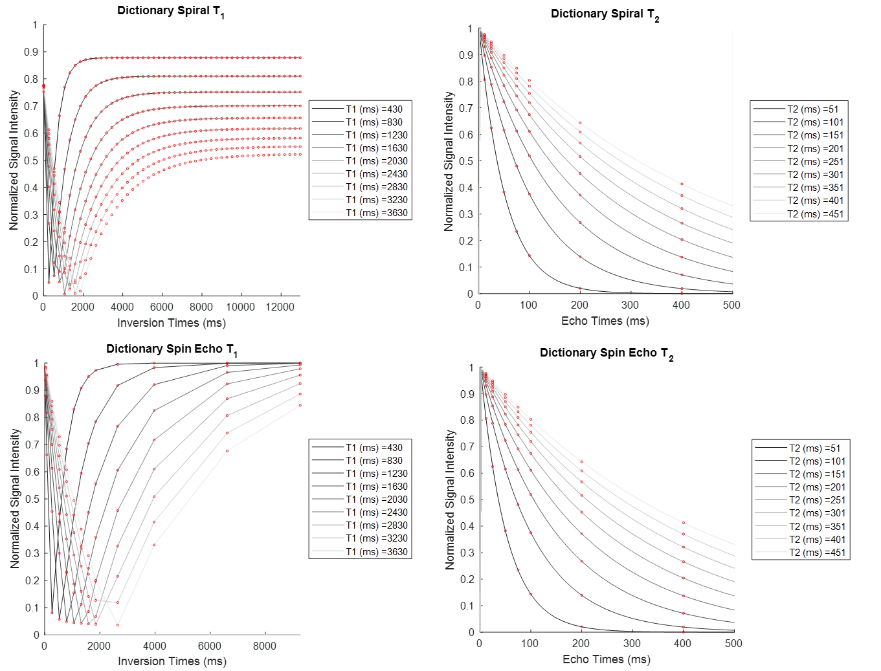


Figure 1 Graphical scheme of the quantitative MRI (qMRI) dictionary development for T_1_ (left) and T_2_ (right) entries, achieved with spiral sequences (top) and spin echo sequences (bottom), and zero-noise simulation. Points in red indicate acquisition times.


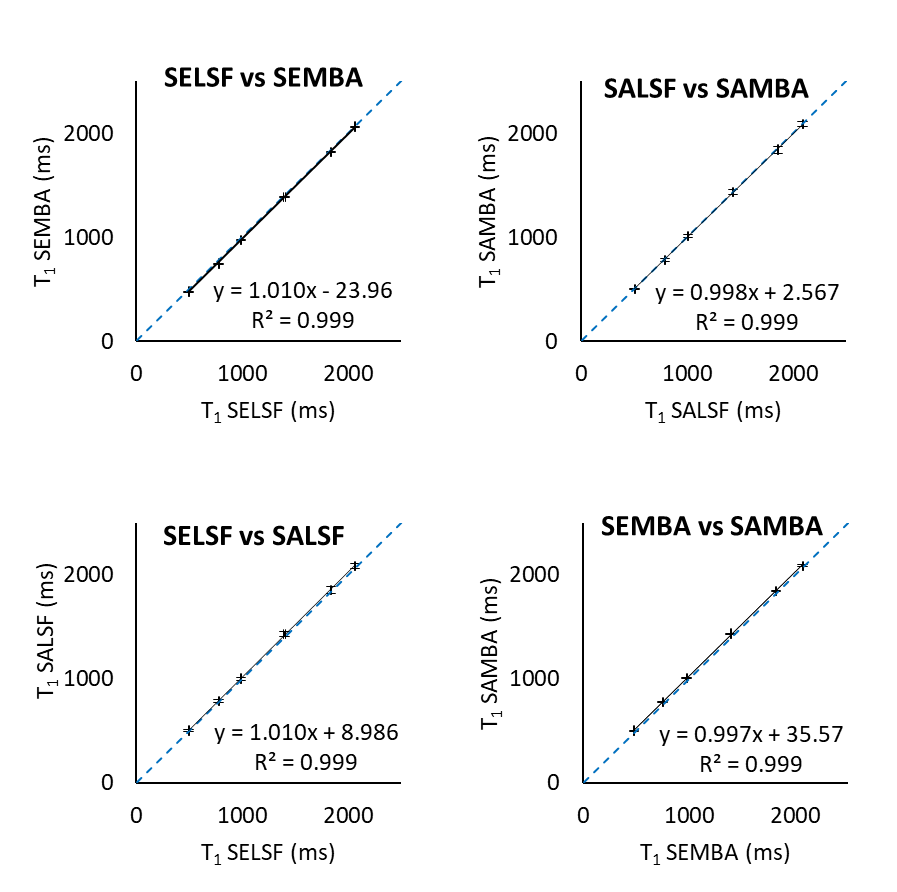


Figure 2 Linear regression comparison of T_1_ measurements acquired and processed with the different schemes: SA = Spiral Acquisition, SE = Spin Echo, MBA = Matching-Based Algorithm, LSF = Least Squares Fitting. Dashed blue line represents unity.


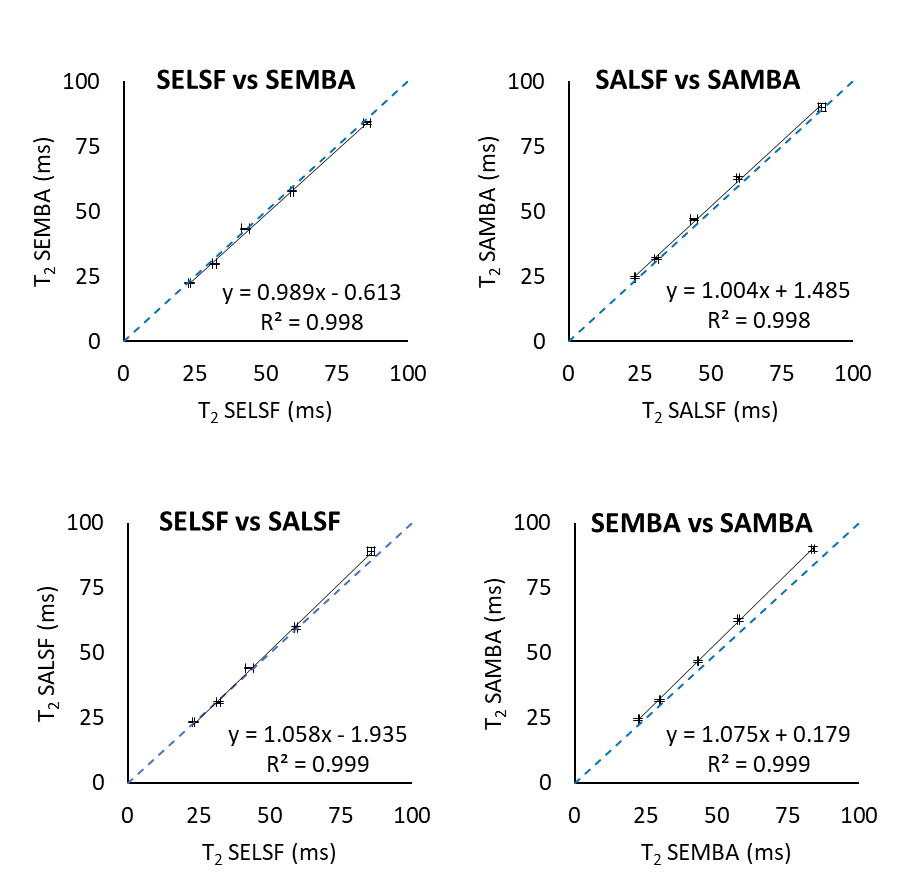


Figure 3 Linear regression comparison of T_2_ measurements acquired and processed with the different schemes: SA = Spiral Acquisition, SE = Spin Echo, MBA = Matching-Based Algorithm, LSF = Least Squares Fitting. Dashed blue line represents unity.


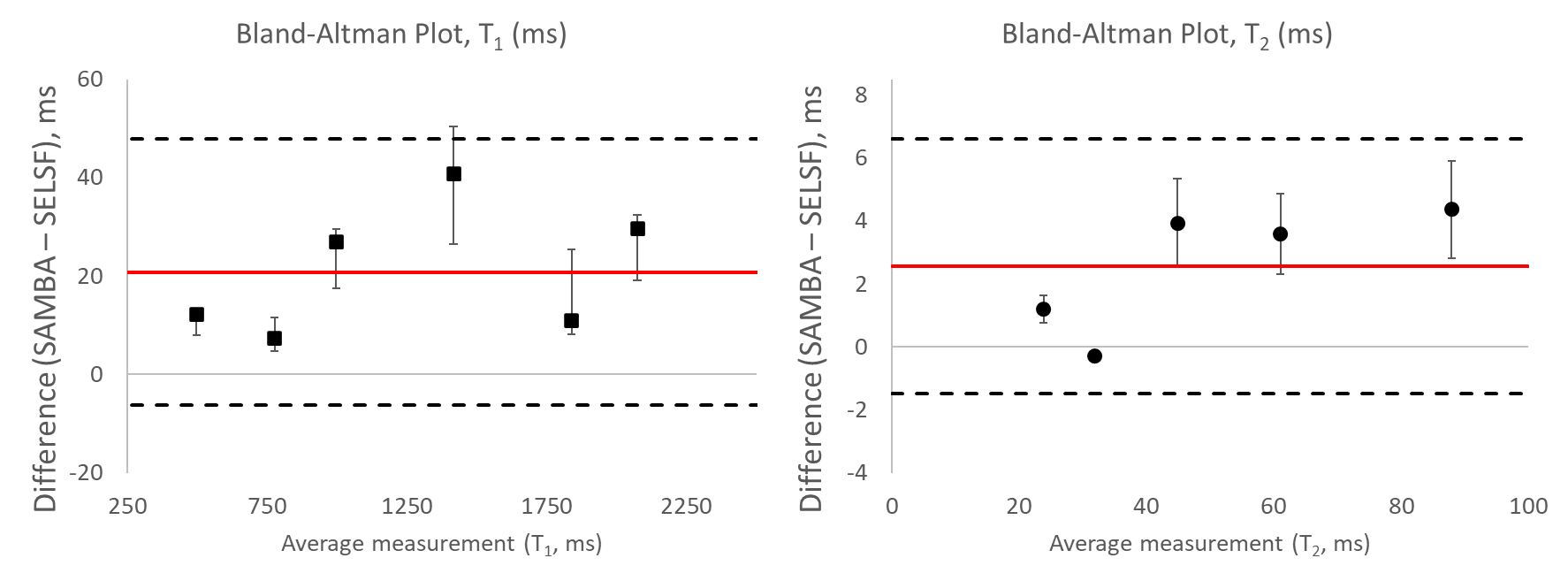


Figure 4 Bland-Altman plots comparison of T_1_ (left) and T_2_ (right) parametric maps calculation between Spin Echo Least Squares Fitting (SELSF) and Spiral Acquisition Matching Based Algorithm (SAMBA) schemes.
